# The M1/M2 Macrophage Polarization and Hepatoprotective Activity of Quercetin in Cyclophosphamide‐Induced Experimental Liver Toxicity

**DOI:** 10.1002/vms3.70183

**Published:** 2025-01-10

**Authors:** Ugur Seker, Emre Uyar, Gul Sahika Gokdemir, Deniz Evrim Kavak, Sevgi Irtegun‐Kandemir

**Affiliations:** ^1^ Department of Histology and Embryology Faculty of Medicine Mardin Artuklu University Mardin Turkey; ^2^ Department of Medical Pharmacology Faculty of Medicine Uskudar University Istanbul Turkey; ^3^ Department of Physiology Faculty of Medicine Mardin Artuklu University Mardin Turkey; ^4^ Department of Medical Biology and Genetics Faculty of Medicine Dokuz Eylul University Izmir Turkey; ^5^ Department of Medical Biology Faculty of Medicine Dicle University Diyarbakir Turkey

**Keywords:** apoptosis, cyclophosphamide, inflammation, liver toxicity, quercetin

## Abstract

**Background:**

Chemotherapy drugs may lead to hepatic injury, which is considered one of the limitations of these drugs.

**Objectives:**

The aim of this study was to evaluate the effect of quercetin (QUE) on M1/M2 macrophage polarization and hepatoprotective effect in cyclophosphamide (CTX)‐induced liver toxicity.

**Methods:**

Twenty‐four mice were divided into four groups (Control, QUE, CTX, CTX + QUE). The CTX and CTX + QUE groups received 200 mg/kg CTX. The animals in the QUE and CTX + QUE groups received 50 mg/kg QUE. All animals were sacrificed, and serum and liver samples were used for laboratory analyses.

**Results:**

Examinations indicated that CTX exposure led to disruption of liver functions and morphological degenerations. Tissue pro‐apoptotic Bax and caspase 3, pro‐inflammatory TNF‐α and IL‐1β, transcription factor NF‐κB, and M1 macrophage polarization marker CD86 were upregulated significant (*p* < 0.05) in this group. In addition, CTX exposure led to significantly (*p* < 0.05) upregulation of the Bax/Bcl‐2 mRNA ratio and DNA fragmentations. The PCNA‐positive hepatic cell ratio and anti‐apoptotic Bcl‐2 expression are remarkably suppressed (*p* < 0.05). Immunohistochemical analyses are also indicated that M2 macrophage polarization marker CD163 is slightly but remarkably (*p* < 0.05) downregulated in the CTX group compared to the Control and QUE groups. The morphological and biochemical disruptions were alleviated in QUE‐treated animals in the CTX + QUE group. Liver function test results, apoptosis, inflammatory, transcription factor NF‐κB, regeneration/proliferation, and apoptotic index results in this group were similar (*p* > 0.05) to the control and QUE groups. The M1 cell surface marker expression of CD86 is significantly (*p* < 0.05) downregulated, and M2 macrophage polarization marker expression of CD163 is upregulated significantly (*p* < 0.05) compared to the CTX group.

**Conclusions:**

This study indicates that QUE has the potential to downregulate CTX‐induced hepatic injury and regulate M1/M2 macrophage polarization to the M2 side, which indirectly demonstrates activation of anti‐inflammatory signalling and tissue repair.

## Graphical abstract

Twenty‐four adult mice were divided into four groups: (1) of six animals each. The animals in CTX and CTX + QUE received 200 mg/kg cyclophosphamide on the 1st and the 7th days of the study (2). The mice in QUE and CTX + QUE received 50 mg/kg of quercetin for 14 days during the study (3). At the end of the study, all animals were sacrificed (4), and blood and liver samples were received (5) for the laboratory analyses (6). Our observations indicated that CTX exposure led to disruption of liver function test results (7). The exposed CTX also increased NF‐κB expression (8) and upmodulation of Bax expression and suppression of anti‐apoptotic Bcl‐2 (9), which activate intracellular caspase 3 activity (10) and DNA fragmentations and downregulation of nuclear PCNA expression (11). This liver degeneration induces the M1/M2 macrophage polarization to the M1 side (12), which induces the production and release of excessive pro‐inflammatory cytokines (13) of TNF‐α and IL‐1β (14) in which the severity of liver organ injury increases (15). In addition, we observed that the administered quercetin significantly influences cyclophosphamide‐induced cellular signalling and organ injury (16) with modulating NF‐κB, thus apoptosis and inflammation‐associated protein expressions, M1/M2 macrophage polarization and DNA fragmentation.

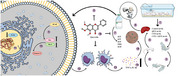

## 1 Introduction

Cyclophosphamide (CTX) is an extensively used antineoplastic drug for veterinary and human medical purposes that is administered in cancers such as chronic and acute leukaemia, lymphoma and myeloma (Mansour and Hasan 2015). It is also used in systemic lupus erythematosus and rheumatoid arthritis due to bearing an immunosuppressive property. The liver is a key organ in CTX metabolism that is regulated through P450 enzymes, and the activation is principally mediated with hydroxylation of four carbon molecules that are neighbouring to the ring, nitrogen (El‐Serafi and Steele 2024). Although it is used in the treatment of plenty of diseases, liver, cardiac and gonadal toxicities, as well as haemorrhagic cystitis and haematopoietic suppression in higher doses, are limiting the benefits of this alkylating drug (Raza and Alghasham 2011; Ye et al. 2022). Under the results of the later clinical and experimental observations, it is demonstrated that CTX may have teratogenic and gamete‐mutating chemical properties (Rengasamy 2017). Oxidative stress‐mediated apoptotic signalling and immune toxicity are believed to be the major reasons for organ dysfunction in high doses of CTX exposure (Cengiz et al. 2020). Upregulation of inflammatory processes is also described as the key mediator in CTX‐induced organ dysfunction (Seker, Kavak, Dokumaci et al. 2024). When the literature in CTX related liver toxicity is considered, most of the studies concluded that the oxidative stress, release of pro‐inflammatory cytokines and upregulation of apoptotic signallings might be controlled through antioxidant drug supplementations (Caglayan et al. 2018). In addition, antioxidant ingredients of plants and synthetic chemicals were reported with beneficial effects on CTX‐induced hepatotoxicity and in other antioxidant supplementation‐required diseases (Singh et al. 2018).

In recent years, targeting the inflammatory and antioxidant signalling systems through regulation of M1/M2 macrophage polarization has been reported as very promising in CTX‐exposed organ injuries and various inflammation‐ and apoptosis‐associated health issues and organ dysfunctions (Li et al. 2022; Sharifnia, Eftekhari, and Mortazavi 2024).

Although in literature there are some previously published articles reporting promising results in the hepatoprotective activity of quercetin in CTX exposure, the limited current literature is not responding to all the questions. The limited preclinical observations also require validation of the observations to forward the applicability of this drug for further purposes. As a strong antioxidant phenolic compound, quercetin is reported with anti‐tumour, anti‐microbial, anti‐inflammatory and anti‐diabetic properties (Iqubal, Syed, Najmi, et al. 2020; Maleki Dana et al. 2021; Najafi et al. 2022). However, as described earlier, the literature based on the hepatoprotective activity on CTX exposure is still very limited, and molecular regulations have not been fully clarified yet. For that reason, in this study, we aimed to examine the hepatoprotective activity of quercetin in high‐dose CTX exposure, with considering M1/M2 macrophage polarization and inflammatory cytokine release, gene and/or protein expression levels of some apoptotic, inflammatory and cell vitality‐associated proteins, DNA fragmentations and liver function test in the last phase.

## 2 Material and Methods

### 2.1 Study Design

Experimental procedures of this study were performed with the approval of the Local Experimental Animal Ethics Committee of Adana Veterinary Control Institute (approval date and number: 05.09.2022–2022–8/486). The obtained mice were equally divided into four groups (Control, QUE, CTX, CTX + QUE). Animals in CTX and CTX + QUE received 200 mg/kg cyclophosphamide (CTX) on the 1st and the 7th days of the experiment. The animals in QUE and CTX + QUE received 50 mg/kg QUE for 14 days. At the end of this study, all mice were sacrificed and blood and liver samples were used for laboratory analyses.

### 2.2 Measurement of Liver Function Tests

The collected blood samples from the animals were centrifuged at ×2000 rpm for 10 min, and the serum was collected for the measurement of alanine transaminase (ALT), aspartate aminotransferase (AST), alkaline phosphatase (ALP), total bilirubin (TBIL) and albumin (ALB) levels. Serum biochemical analyses were performed with the ADVIA 1800 Clinical Chemistry Analyser System (Siemens Healthineers, Erlangen, DE) and the measurements are considered statistically. The results of AST, ALT and ALP are expressed as U/L; the TBIL levels were expressed as mg/dl and ALB levels are shown as g/dL.

### 2.3 Measurement of Tissue TNF‐α and IL‐1β Levels

The liver tissues were homogenized using a homogenizer (NF 800 R, NUVE, Ankara) in 50 mmol/L phosphate buffer (pH 7.4). Homogenates were centrifuged at 4°C at 15,000 × *g* for 15 min, and supernatants were stored at −80°C for further examinations. The concentrations of TNF‐α and IL‐1β were performed using an enzyme‐linked immunosorbent assay (ELISA) according to the manufacturer's directions (Elabscience Biotechnology; Wuhan, China). The concentrations were normalized by liver weight used for protein homogenization. The optical densities were analysed using a UV/VIS spectrometer (Shimadzu 1601, Kyoto, Japan), and the results were expressed as pg/dL.

### 2.4 Tissue Processing and H&E Staining

Formalin‐fixed tissue samples were embedded into paraffin blocks following a routine tissue processing protocol (Ayaz et al. 2023). Five µm‐thick sections were received from paraffin‐embedded liver tissue samples with a rotary microtome, and the samples were stained with haematoxylin and eosin for pathological examinations. The rest of the serial sections were used for immunohistochemistry and TUNEL Assay.

### 2.5 Immunohistochemistry

The immunohistochemistry of apoptosis associated Bcl‐2‐associated X protein (Bax; Santa Cruz Biotechnology; dilution ratio: 1/100; catalog no: sc‐7480), B‐cell lymphoma 2 (Bcl‐2; Santa Cruz Biotechnology; dilution ratio: 1/50; catalog no: sc‐7382) and cysteine–aspartic acid protease 3 (caspase 3; Santa Cruz Biotechnology; dilution ratio: 1/100; catalog no: sc‐56053), pro‐inflammatory tumour necrosis factor alpha (TNF‐α; Santa Cruz; dilution ratio: 1/300; catalog no: sc‐52746), proliferative nuclear antigen (PCNA; Thermo Scientific, Waltham, MA, USA; dilution ratio: 1/150; catalog no: sc‐ 25280), transcription factor nuclear factor kappa‐light‐chain‐enhancer of activated B cells (NF‐κB; Santa Cruz Biotechnology; dilution ratio: 1/150; catalog no: sc‐8008), M1 and M2 macrophage polarization surface markers of CD86 (Santa Cruz Biotechnology; dilution ratio: 1/50; catalog no: sc‐28347) and CD163 (Santa Cruz Biotechnology; dilution ratio: 1/50; catalog no: sc‐58965) were performed with routine immunohistochemistry protocols (Kurt et al. 2023). Briefly, the deparaffinized, rehydrated sections were exposed to antigen retrieval and blocking of non‐specific binding with incubation in 3% H_2_O_2_ and BSA‐based blocking buffer. The secondary antibody, enzyme and chromogenic reaction were developed through a ready‐to‐use IHC kit (large‐volume detection system: anti‐Polyvalent, HRP; Thermo Scientific, Waltham, MA, USA; catalog no: TP‐125‐HL) and DAB chromogen substrate system (DAB Plus Substrate Staining System; Thermo Scientific, Waltham, MA, USA; catalog no: TP‐125‐HL). All steps of the kits were performed according to the directions of the manufacturer.

### 2.6 TUNEL Assay Protocol

Terminal deoxynucleotidyl transferase dUTP nick end labelling (TUNEL Assay) was also performed on the sections, and a commercially produced in situ cell death detection kit, fluorescein (Cat. No: 11684795910, Roche, DE), was used for detection of the double‐stranded DNA breaks in liver tissues. All steps in TUNEL Assay were performed according to the manufacturer's recommendations and as described previously. The samples were counterstained with DAPI antifade media and examined under a fluorescein‐attached light microscope (Seker, Kavak, Guzel, et al. 2022).

### 2.7 Real‐Time Quantitative PCR

The mRNA expression of apoptosis‐related genes (Bax and Bcl‐2) was quantified by RT‐qPCR (Seker, Kavak, Dokumaci, et al. 2024). The GeneAll hybrid‐RTM blood RNA extraction kit (Cat no. 305–101, GeneAll, Seoul, KR) was used to isolate total RNA from tissues according to the manufacturer's protocols. The RNA quality parameters recommended for RT‐qPCR analysis were measured by μDrop Plate MultiSkan spectrophotometer, and an A260/280 ratio of 1.8–2.0 and an A260/A230 ratio greater than 1.8 were considered acceptable. The cDNA was generated using total RNA and synthesized with ProtoScript II Reverse Transcriptase (Cat no: M0368S, New England Biolabs, MA, USA). All qPCRs were performed in triplicate on the LightCycler FastStart DNA MasterPLUS SYBR Green I (Cat no: 03515885001, Roche Life Science, Basel, CH). The DNA samples (10 ng) were added into 2.5 µL of the reaction mixture for a final volume of 10 µL per reaction. The cycling conditions were 95°C for 10 min (denaturation and Taq polymerase activation), followed by a 45‐cycle amplification program consisting of 95°C for 10 s, 58°C for 5 s and 72°C for 4 s. The amplification program was followed by a melting cycle of 95°C for 0 s, 62°C for 15 s and slow heating to 95°C for 0 s, with a transition rate of 20°C/s. Quantitative measurements were conducted using a LightCycler RT‐qPCR equipment with LightCycler 480 relative quantification software. Hypoxanthine guanine phosphoribosyl transferase (HPRT) was used as a housekeeping control gene for normalization of the target genes before statistical analysis was performed. The results were expressed as the target/reference ratio of each sample normalized by the target/reference ratio of the calibrator (1). The primer sequences used for RT‐qPCR are shown in Table 1.

**TABLE 1:** Forward and reverse primers of housekeeping Hprt, Bax and Bcl‐2 sequences used in this study for RT‐qPCR.

Name of the gene	Abbr.	Primer sequence (5′‐3′)	Size (bp)	Accession vers.
Hypoxanthine guanine phosphoribosyl transferase	Hprt	F: 5′ GTG GAG ATG ATC TCT CAA CT 3′ R: 5′ ACA TGA TTC AAA TCC CTG AAG 3′	210	NM_013556.2
Bcl‐2‐associated X protein	Bax	F: 5′ AAG AAG CTG AGC GAG T 3′ R: 5′ GCC CAT GAT GGT TCT G 3′	246	NM_001411995.1
B‐cell lymphoma 2	Bcl‐2	F: 5′ ACC TGA CGC CCT TCA C 3′ R: 5′ AGG TAC TCA GTC ATC CAC 3′	184	NM_009741.5

### 2.8 Statistical Analyses

One‐way ANOVA test was used for statistical consideration, and the multiple comparisons were performed with a post‐hoc Tukey test. The results were considered as significant if *p* < 0.05 and the results were expressed as mean ± standard deviation (SD).

## 3 Results

### 3.1 Serum Biochemical Analysis Results

Serum ALT, AST, ALP and TBIL levels were significantly (*p* < 0.05) upregulated in the CTX group compared to control and QUE groups (Table 2). These changes were improved in the CTX + QUE group compared to the CTX group. Results in the CTX + QUE group was similar (*p* > 0.05) to the control and QUE groups. We observed a significant downregulation of ALB (*p* < 0.05) in the CTX group compared to the control and QUE groups. However, the serum ALB level in the CTX + QUE group was similar to the CTX group (*p* > 0.05).

**TABLE 2:** Liver function test results in groups. All measurements were evaluated statistically with one‐way ANOVA and post‐hoc Tukey test.

	ALT (U/L)	AST (U/L)	ALP (U/L)	TBIL (md/dL)	ALB (g/dL)
**Control**	78.40 ± 26.90^a^	165.20 ± 73.19^a^	120.60 ± 27.56^c^	0.04 ± 0.03^g^	4.38 ± 0.16^c^
**QUE**	84.60 ± 15.90^a^	154.40 ± 44.75^a^	123.20 ± 23.66^c^	0.06 ± 0.02^f,g^	4.30 ± 0.16^c^
**CTX**	131.60 ± 11.74^b^	309.00 ± 48.21^b^	159.80 ± 6.26^d^	0.11 ± 0.02^e^	3.60 ± 0.69^d^
**CTX + QUE**	104.60 ± 19.27^a,b^	229.20 ± 57.33^a,b^	140.80 ± 15.32^c,d^	. 0.08 ± 0.01^e,f^	4.18 ± 0.22^c,d^

*Note*: Different superscripts indicate statistically significance between the groups ^a,b^ *p *< 0.001, ^c,d^ *p* < 0.05, ^e,f^ *p* < 0.05, ^e,g^ *p *< 0.001, ^f,g^ *p* < 0.05.

### 3.2 Tissue TNF‐α and IL‐1β Results

Analyses indicated that the highest TNF‐α was observed in the CTX group, and results of this group were significantly higher (*p* < 0.01) than control, QUE and CTX + QUE groups. The tissue TNF‐α level in control, QUE, and CTX + QUE groups was statistically similar (*p* > 0.05) to each other. When tissue level of IL‐1β is evaluated, our measurements indicated that the most up‐regulated IL‐1β is in the CTX group. Results of this group were significantly different than the control, QUE (*p* < 0.01) and CTX + QUE (*p* < 0.05) groups. IL‐1β levels in control and QUE are similar (*p* > 0.05) to each other, but results in CTX + QUE were significantly different from control (*p* < 0.05) and QUE (*p* < 0.01) groups. Results of the statistical analyses are shown in Table 3.

**TABLE 3:** Results of the statistical analyses of tissue TNF‐α and IL‐1β.

	Tissue TNF‐α level (ng/mL)	Tissue IL‐1β level (ng/mL)
**Control**	12.09 ± 1.77^a^	11.53 ± 0.20^d^
**QUE**	11.75 ± 2.68^b^	10.04 ± 2.41^e^
**CTX**	17.60 ± 2.82^a^	17.53 ± 4.05^c^
**CTX + QUE**	12.55 ± 1.84^a^	15.01 ± 1.72^c,d,e^

*Note*: Different superscripts indicate statistically significance between the groups. ^a,b^ *p* < 0.05, ^c,d^ *p* < 0.05, ^c,e^ *p* < 0.01, ^d,e^ *p *> 0.05.

### 3.3 Histopathological Results

**FIGURE 1:**
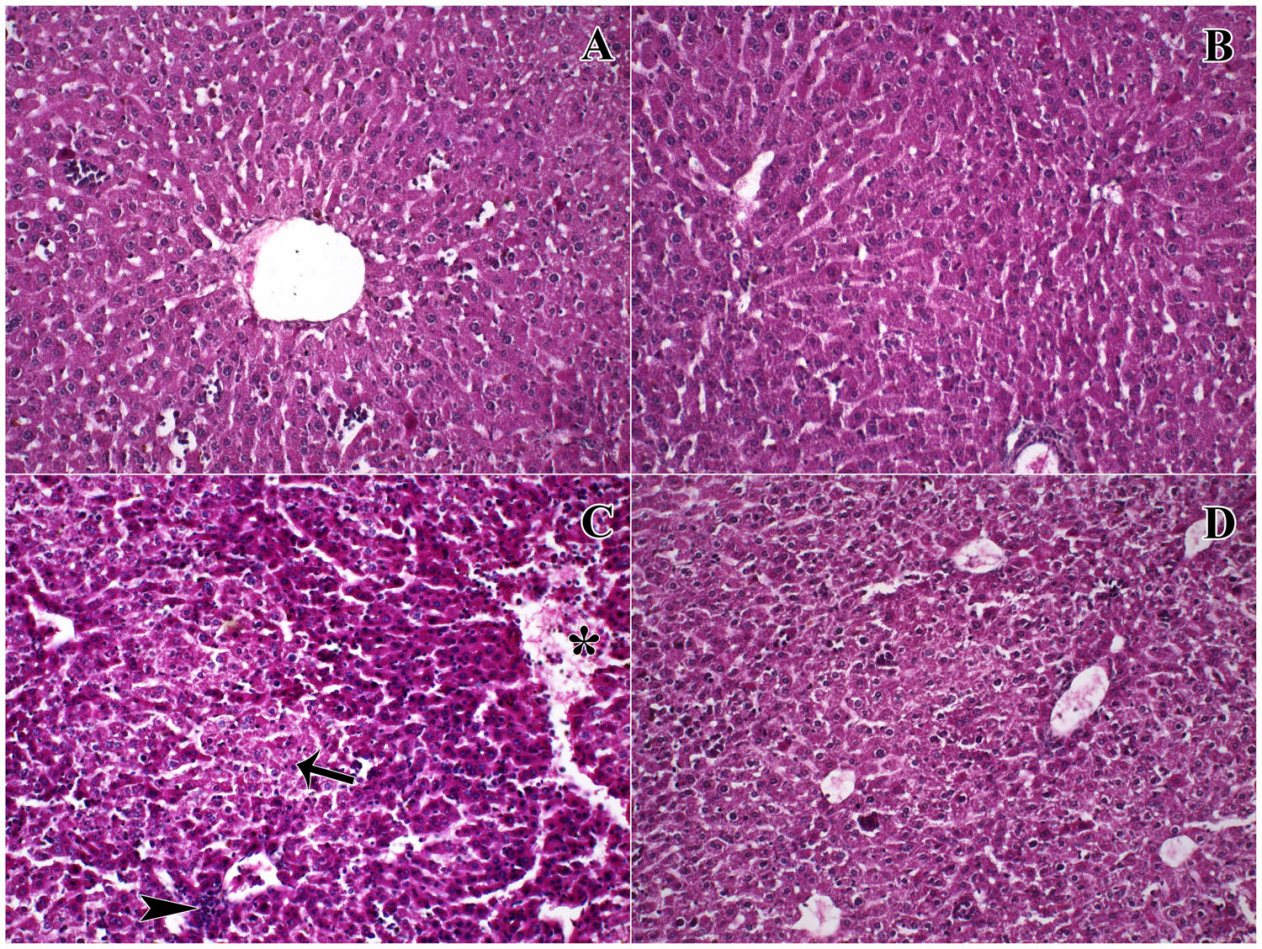
Representative micrographs of the groups. Irregular and oedematous parenchyma (*), increased pyknotic cell nuclei (arrow), and increased inflammatory cell accumulation in the CTX group. Pathological changes significantly improved in QUE‐treated animals. A: Control, B: QUE, C: CTX, D: CTX + QUE. Staining: Haematoxylin and eosin. Magnification: 40×.

We observed normal liver histology in the sections of the control and QUE groups, but there were severe pathological alterations such as increased oedematous parenchyma within the hepatic lobules and widespread nuclear pyknosis in hepatocytes (Figure 1). Hepatocytes were disrupted, and cell shapes were irregular in this group. We observed desquamated endothelial cells in the cavity of the central vein, and the shape of the central vein, was irregular compared to the control and QUE groups. The structural irregularities were also observed at the sinusoidal space of this group. The mentioned pathological observations were alleviated, and some of the pathological changes were almost disappeared, but sinusoidal irregularities were still obvious, and parenchymal oedema was still observable, although it was significantly improved in quercetin‐treated animals.

### 3.4 Immunohistochemistry Results

**FIGURE 2:**
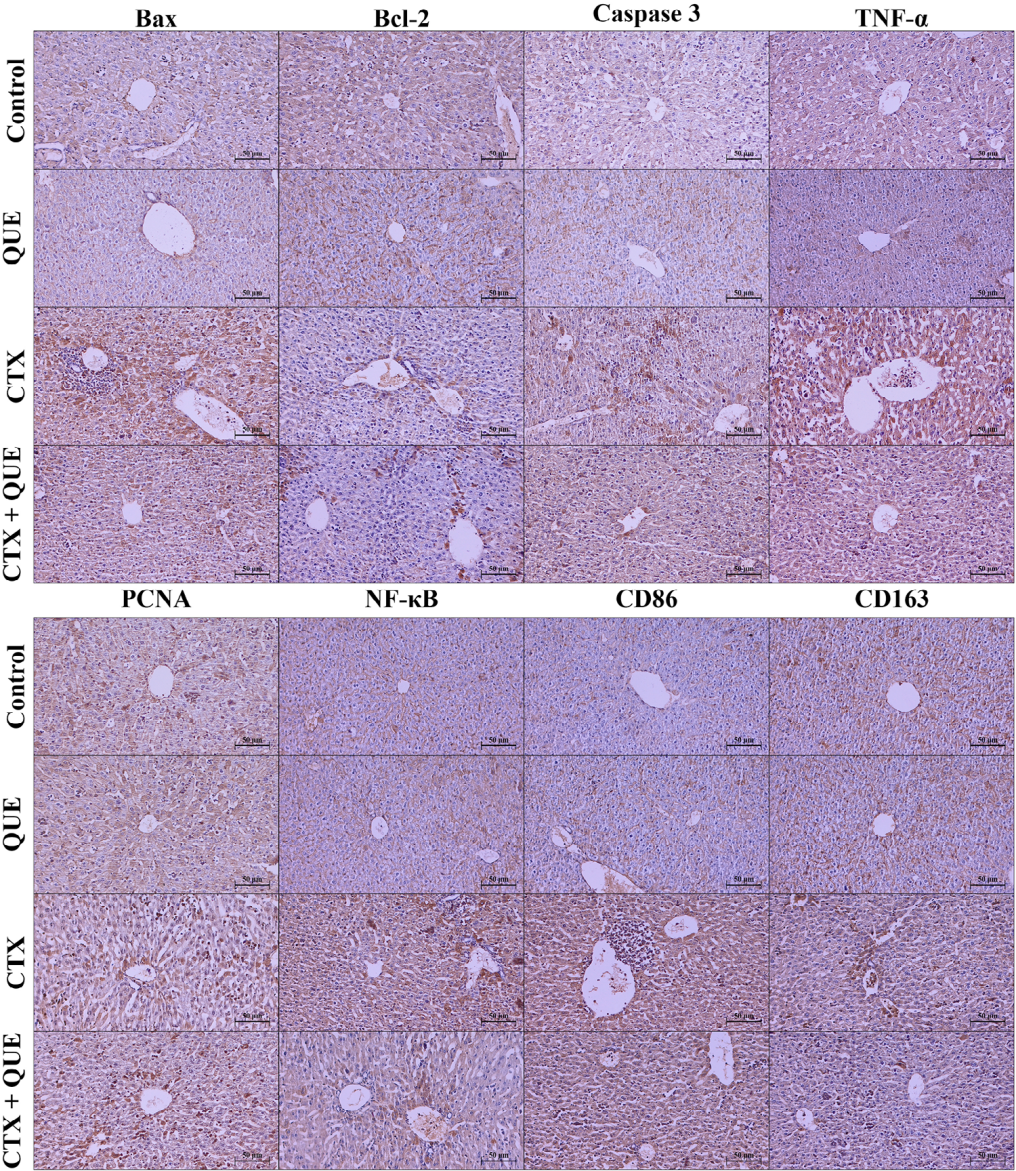
Representative immunohistochemistry micrographs of Bax, Bcl‐2, caspase 3, TNF‐α, PCNA, NF‐κB, CD86 and CD163. Brown staining indicates the immunopositivity and density of the protein of interest. The sections were counterstained to determine the immunodensity of the interest protein. Counterstaining: Haematoxylin. Bar: 50 µm.

Representative immunohistochemistry micrograph is shown in Figure 2. Our immunodensity analyses demonstrated that CTX exposure led to a significantly (*p* < 0.01) increase of Bax, caspase 3 and pro‐inflammatory TNF‐α compared to control and QUE groups. Anti‐apoptotic Bcl‐2 and proliferation/regeneration marker PCNA levels are significantly (*p* < 0.05 and *p* < 0.01 respectively) downregulated in the CTX group compared to the control and QUE groups. The transcription factor NF‐κB level is significantly increased in the CTX group compared to the control (*p* < 0.05) and QUE (*p* < 0.01) groups. While the M2 macrophage polarization marker CD163 is significantly (*p* < 0.05) reduced, the M1 macrophage polarization marker of CD86 expression is increased significantly (*p* < 0.01) in the CTX group compared to the control and QUE groups. When the immunodensity analyses are considered, the Bax and NF‐κB levels in the CTX + QUE group were significantly different (*p* < 0.05) from the CTX group but similar to both control and QUE groups. The PCNA‐positive cell ratio in the CTX + QUE group was significantly different (*p* < 0.05) from CTX, control and QUE groups. Tissue Bcl‐2, caspase 3 and TNF‐α levels in the CTX + QUE group were similar (*p* > 0.05) to the CTX, control and QUE groups. When the M1/M2 macrophage markers were evaluated, the CD86 level in the CTX + QUE group was significantly different (*p* < 0.05) from the CTX group, but the results of CD86 were similar (*p* > 0.05) to the control and QUE groups. In addition, CD163 immunodensity analyses demonstrated the similarity of the CTX + QUE group (*p* > 0.05) with control and QUE groups. However, the immunodensity of CD163 in CTX + QUE group was significantly different (*p* < 0.05) from the CTX group (Table 4).

**TABLE 4:** Results of the statistical analyses of immunodensity analyses, PCNA positive hepatocyte ratio and Apoptotic index through DNA fragmentation in hepatocytes.

Immunodensity and positivity (%)	Control	QUE	CTX	CTX + QUE	*p* Value
**Bax**	32.74 ± 4.91^a,b^	27.81 ± 3.57^a^	43.79 ± 9.19^c^	37.43 ± 9.24^b^	^a,b^ *p* < 0.01, ^a,c^ *p *< 0.01, ^b,c^ *p* < 0.05
**Bcl‐2**	29.08 ± 4.13^a^	29.6 ± 4.72^a^	24.43 ± 6.35^b^	27.09 ± 4.95^a,b^	^a,b^ *p* < 0.05
**Caspase‐3**	33.87 ± 3.44^a^	32.57 ± 3.15^a^	42.90 ± 9.54^b^	37.85 ± 8.44^a,b^	^a,b^ *p* < 0.01
**TNF‐α**	16.30 ± 3.08^a^	15.92 ± 2.39^a^	21.27 ± 5.52^b^	18.06 ± 3.98^a^	^a,b^ *p *< 0.01
**PCNA**	84.83 ± 6.34^a^	85.83 ± 6.83^a^	36.48 ± 7.86^c^	58.80 ± 11.99^b^	^a,b^ *p* < 0.01, ^a,c^ *p *< 0.01, ^b,c^ *p* < 0.01
**NF‐κB**	27.06 ± 5.20^b^	26.25 ± 6.50^a^	36.62 ± 11.94^c^	27.55 ± 11.36^b^	^a,b^ *p > *0.05, ^a,c^ *p < *0.01, ^b,c^ *p* < 0.05
**CD86**	9.61 ± 2.75^a^	8.96 ± 2.9^a^	21.81 ± 12.17^c^	14.8 ± 7.78^b^	^a,b^ *p > *0.05, ^a,c^ *p* < 0.01, ^b,c^ *p* < 0.05
**CD163**	10.23 ± 3.16^a^	9.97 ± 2.41^a^	7.47 ± 3.06^b^	9.91 ± 2.26^a^	^a,b^ *p* < 0.05
**Apoptotic** **index**	2.39 ± 1.09^a,b^	1.93 ± 0.80^a^	7.51 ± 3.43^c^	4.24 ± 3.00^b^	^a,b^ *p* < 0.05, ^a,c^ *p *< 0.01, ^b,c^ *p* < 0.01

*Note*: Different superscripts on the results at the same column demonstrate statistically significance.

### 3.5 DNA Fragmentation (TUNEL Assay) Results

**FIGURE 3:**
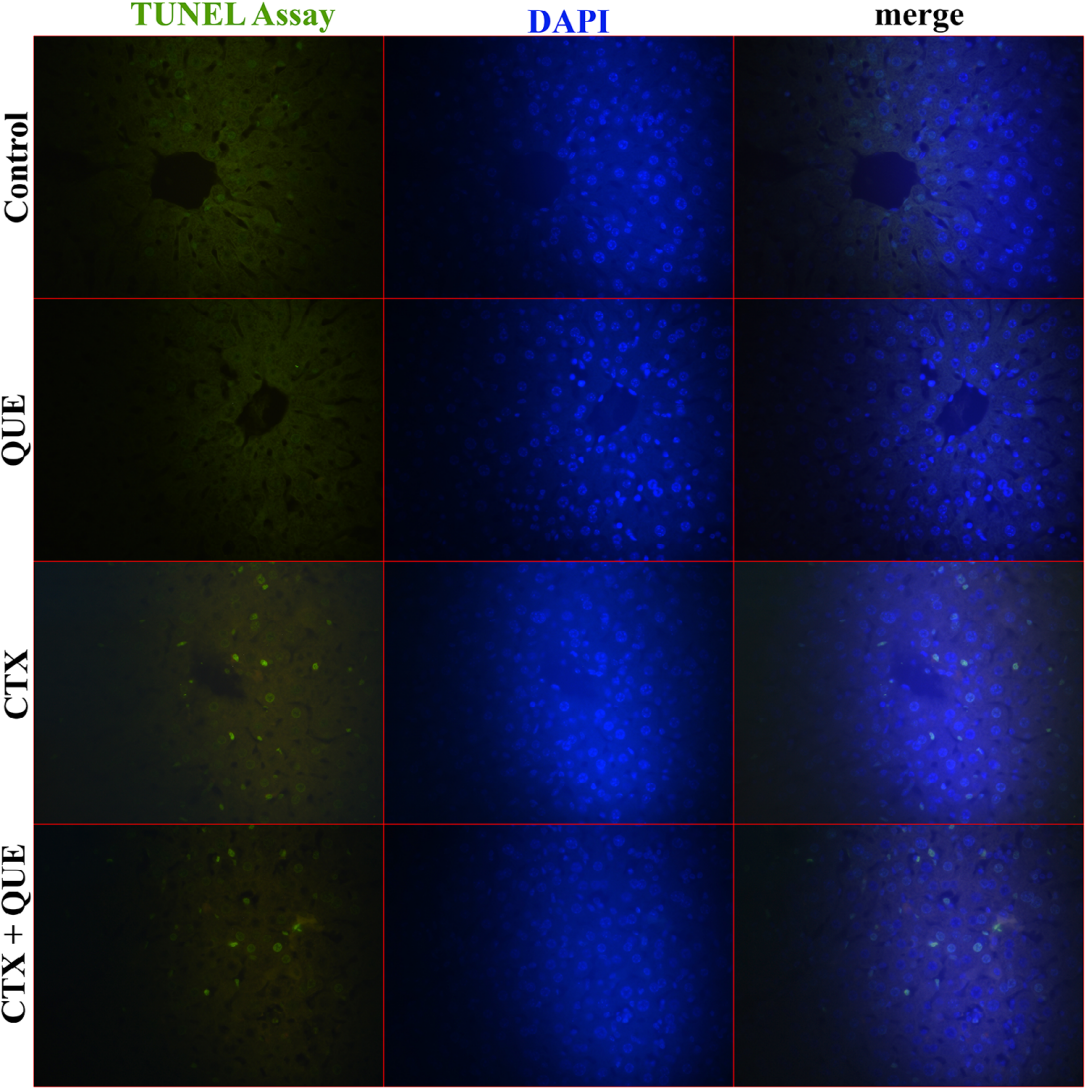
Representative micrographs of TUNEL Assay of the groups. Green fluorescence nuclei indicate double‐strand DNA break. All nuclei were counterstained with DAPI, and the micrographs were merged to obtain distribution of DNA fragmented hepatocyte ratio over total nuclei in hepatic lobules. Staining: TUNEL Assay (fluorescein), Counterstain: DAPI, Magnification: 40×.

Results of analyses indicate that CTX exposure significantly increased (*p* < 0.01) DNA fragmentation compared to Control and QUE groups. The apoptotic index was significantly improved (*p* < 0.01) in quercetin‐treated animals of the CTX + QUE group compared to the CTX group. However, the results in the CTX + QUE group were significantly higher than QUE (*p* < 0.05) but similar to (*p* > 0.05) the control group. Fluorescein TUNEL Assay micrographs are shown in Figure 3.

### 3.6 Bax/Bcl‐2 mRNA Ratio Results Through RT‐qPCR

**FIGURE 4:**
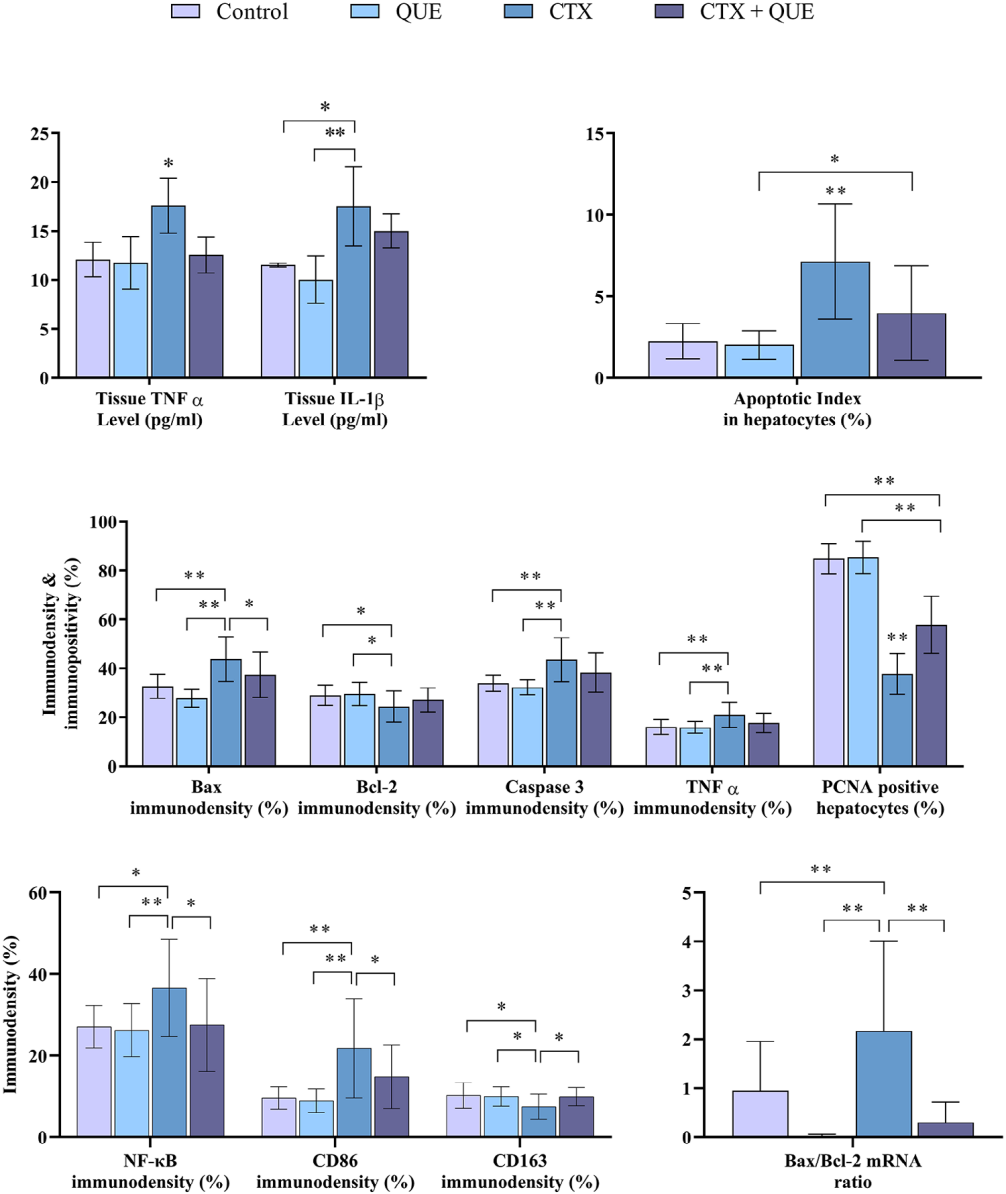
Graphical demonstration of the statistical analysis of tissue TNF‐α, IL‐1β levels of ELISA Assay, tissue immunodensity of Bax, Bcl‐2, caspase 3, TNF‐α, positivity of PCNA and apoptotic index among the groups. In addition to, graphs indicate statistically significance of Bax/Bcl‐2 mRNA ratio among the groups. Different superscripts on each or between the columns indicate differences between the groups. *p < 0.05, **p < 0.01.

In our study, we evaluated the potential protective effect of CTX‐induced toxicity through quercetin assessing the apoptotic mRNA expression Bax/Bcl‐2 ratio. The mean Bax/Bcl‐2 ratio was 0.978 ± 0.031 in the control group, 0.028 ± 0.004 in the QUE group, 2.001 ± 0.245 in the CTX group and 0.362 ± 0.083 in the CTX + QUE group. The statistically significant lower Bax/Bcl‐2 ratio in the quercetin group compared to the control and CTX groups (*p* ≤ 0.01). In the chemotherapeutic agent CTX group, the Bax/Bcl‐2 ratio was found to be approximately two times higher than in the control group (*p* ≤ 0.01). But, in the combination of CTX with QUE, the Bax/Bcl‐2 ratio was significantly lower than in the CTX group (*p* ≤ 0.001). A graphical demonstration of the RT‐qPCR fold change for the Bax/Bcl‐2 ratio mRNA level is shown in Figure 4.

## 4 Discussion

The most observed side effects of chemotherapy exposure are hypertension, headache, vomiting, and high doses may result in organ dysfunction that may be severe as much as life‐threatening. In recent years, researchers have been performing studies to explore the potential protective activity of natural antioxidants on organs because of the wide use of these substances in clinical applications and experimental studies (Khan, Afaq, and Mukhtar 2008). CTX is one of the first discovered chemotherapy drugs, which is already used in immune suppression required for severe forms of disorders such as systemic sclerosis, vasculitis, systemic lupus erythematosus, myopathies, rheumatoid arthritis (Arnaud et al. 2017; Brock and Wilmanns 1958). Although CTX is considered one of the most beneficial anti‐cancer drugs, high doses over 120 mg/kg may result in multi‐organ toxicity, including the liver, where pre‐drug CTX is converted into its activated form, 4‐hydroxycyclophosphamide (De Jonge et al. 2005; Emadi, Jones, and Brodsky 2009). Experimental studies reported that CTX exposure leads to hepatic parenchyma and sinusoidal degenerations, even pyknotic cell nuclei, which are important proof of apoptotic cell death (Elshater et al. 2018). Microscopic examinations of previously performed experimental studies also indicated that repetitive administration of low doses also tends to lead to hepatic degenerations and pathological alterations (Cuce et al. 2015). Observations indicated that the liver damage in CTX exposure is a result of improved oxidative stress (specially elevated MDA level in liver tissue), liver function enzymes of ALT and AST, but downregulation of endogenous GSH level (Cengiz et al. 2022). Furthermore, pre‐clinical studies indicated that CTX‐induced hepatotoxicity is also related to increased tissue expression levels of apoptotic caspase 3, inflammatory TNF‐α and IL‐1β (Caglayan et al. 2018). Even it has also been reported that the administration of 200 mg/kg CTX significantly increases DNA fragmentation in the hepatic tissue of experimental animals (Ma et al. 2021). The authors of this study also indicated that inhibition of Txnip/Trx/NF‐κB signalling improves liver injury and apoptotic cell death in parenchyma. When the literature is considered as a whole, it's possible to reach the conclusion that numerous signalling pathways are interfering with the hepatotoxic process of CTX. For example, Aladeilah et al. indicated that CTX‐induced hepatotoxicity can be controlled with antioxidant administration through upregulation of Nrf2/HO‐1 signalling (Aladaileh et al. 2019). In another study, Iqubal et al. reported that the antioxidant profile‐bearing drugs alleviate hepatotoxicity in experimental animals with modulatory effects on Nrf2, NF‐κB p65 and caspase 3 signalling (Iqubal, Syed, Ali, et al. 2020). For that reason, it's obvious that the hepatotoxic effect of CTX is a very complicated process, and numerous researchers around the world are still aiming to explore this mystery. On the other hand, scientists are still looking for the most successful hepatoprotective drug to handle the success of high doses of CTX. While the studies based on the hepatoprotective effect of quercetin on liver toxicity are very limited, there is a recently published article reporting that CTX‐induced hepatotoxicity leads to the dramatic upregulation of pro‐apoptotic caspase 3 and Bax, pro‐inflammatory IL‐1β and TNF‐α immunoexpression, and even the formation of hepato‐pathological conditions such as fibrosis (Turedi 2023). This study also indicated that quercetin treatment alleviated these liver dysfunction effects of CTX. In terms of these observations, our results are consistent with this study, and quercetin treatment in CTX administrations is providing promising results. Our observations are not only consistent with the apoptosis and inflammatory process regulatory effect of quercetin in CTX‐induced hepatic injury but also indicate that both gene and protein expression processes are regulated throughout this process. Moreover, our results strongly demonstrate the potential role of M1/M2 macrophage polarization on the hepatotoxicity effect of CTX through driving the polarization process to the M1 side, but the valuable interference of quercetin treatment on this process and regulated polarization of macrophages to the M2 side.

The role of M1/M2 macrophage polarization may change depending on the source of the activation. However, most of the studies indicated that M1 macrophage polarization is directly associated with the inflammatory process, and the M2 polarization is a key process in anti‐inflammatory response and tissue remodelling and repair (Barros et al. 2013). The role of M1/M2 macrophage polarization in chemotherapy‐associated treatments and toxicity is still not well known, but a previously published article indicated that quercetin treatment inhibits M1 macrophage polarization but activates alternative M2 polarization which indicates this flavonol's influence on macrophage polarization and the inflammatory process (Tsai et al. 2021). Although the current literature associated with CTX, quercetin and M1/M2 macrophages is very limited, a previously published article demonstrated that chemotherapy drugs of doxorubicin regulate M1/M2 macrophage polarization to the inflammatory direction, but antioxidant drug treatments regulate this process and provide an anti‐inflammatory response with upmodulating M2 macrophage activity (Zhang et al. 2020). This study indicates the significance of M1/M2 macrophage polarization in chemotherapy drug administrations and understanding the role of chemopreventive drugs.

While reviewing the literature, we have not reached any study that considers the signalling of proliferation‐regulating proteins such as PCNA in CTX‐induced hepatotoxicity and any protective drug treatment. For that reason, our observations indicated this co‐treatment supported the regeneration profile of live in CTX‐induced hepatic toxicity, which is demonstrated through a reliable marker of liver proliferation and regeneration, the PCNA protein expression levels (Dong et al. 2024). In a previously published study, it was reported that quercetin administration in CTX exposure reduced pathological alterations and oxidative stress in the lung tissue of experimental animals (Şengül et al. 2017). The results of this study demonstrated that quercetin is a novel drug treatment regulating MDA, GSH and SOD levels. As we described earlier, the current literature focuses on the quercetin's potent hepatoprotective activity on CTX‐induced liver toxicity, is very limited except for a recently published but promising results are reported by Kocahan et al. (Kocahan et al. 2017). Authors of this study reported that low doses of CTX upregulated oxidative stress via effecting the tissue level of oxidant and antioxidant enzyme systems. Results of this study also indicated that quercetin treatment improved these biochemical alterations. In another experimental study, Doustimotlagh et al. reported that upregulated oxidative stress was alleviated in quercetin‐treated animals isolated from *Nasturtium officinale R. Br*. (Doustimotlagh et al. 2020). Similarly, Naqvi et al. reported that quercetin‐loaded nanoparticles were found successful in reducing the adverse effects of CTX in terms of oxidative stress, liver enzyme levels and histopathological morphology (Naqvi, Sharma, and Flora 2019).

## 5 Conclusions

In conclusion, our experiment indicated that CTX exposure leads to severe hepatotoxicity with disrupted liver function test results, increased pro‐apoptotic caspase 3 expression and pro‐inflammatory cytokine, and DNA fragmentation, but reduced of PCNA. CTX exposure also resulted in the upmodulation of CD86 expression which is an important sign of macrophage polarization to the M1 side. However, quercetin treatment improves pathological alterations and modulates apoptosis, inflammation and regeneration/proliferative cell protein expressions. One of the most important effects we observed is that the CTX‐associated activation of M1 macrophage polarization is inhibited, and the CD163 expression is upmodulated, which is a significant marker for the M2 macrophage polarization. Although we observed very promising results in quercetin treatment during CTX‐exposed hepatic toxicity, there are some limitations that should be addressed. First of all, we have not examined pro‐survival signalling pathways such as Akt‐PI3K‐mTOR or other kinases, transcription factors and growth factors. In addition, the possible dose‐ and time‐dependent effect of quercetin in CTX‐induced hepatic protection process is another limited side of this study. For that reason, we believe more studies with examinations of more cellular processes are required to evaluate and understand the cellular response of hepatic tissue to CTX‐induced toxicity and the protective activity of quercetin.

## Author Contributions

**Ugur Seker**: conceptualization, methodology, investigation, data curation, supervision, resources, project administration, writing–original draft, validation, visualization, funding acquisition, software, writing–review and editing. **Emre Uyar**: methodology, data curation, validation, writing–original draft, formal analysis, funding acquisition, software, writing–review and editing. **Gul Sahika Gokdemir**: data curation, methodology, writing–original draft, writing–review and editing. **Deniz Evrim Kavak**: Investigation, data curation, formal analysis. **Sevgi Irtegun‐Kandemir**: investigation, data curation.

## Ethics Statement

Experimental procedures of this study were performed with the approval of the Local Experimental Animal Ethics Committee of Adana Veterinary Control Institute (approval date and number: 05.09.2022–2022‐8/486).

## Conflicts of Interest

The authors declare no conflicts of interest.

### Peer Review

The peer review history for this article is available at https://publons.com/publon/10.1002/vms3.70183

## Data Availability

The data that support the findings of this study are available from the corresponding author upon reasonable request.
